# Wheat‐ghretropins: novel ghrelin‐releasing peptides derived from wheat protein

**DOI:** 10.1002/2211-5463.13124

**Published:** 2021-03-18

**Authors:** Kana Tanikawa, Kentaro Kaneko, Shimon Abe, Jyunya Nakato, Yuki Tokuyama, Sayano Odaka, Hiroshi Iwakura, Masaru Sato, Atsushi Kurabayashi, Hideyuki Suzuki, Miki Makita, Hiroyuki Ikemoto, Shigenobu Matsumura, Kazuo Inoue, Kousaku Ohinata

**Affiliations:** ^1^ Division of Food Science and Biotechnology Graduate School of Agriculture Kyoto University Uji Japan; ^2^ The First Department of Medicine Wakayama Medical University Japan; ^3^ Department of Applied Genomics Kazusa DNA Research Institutes Kisarazu Japan; ^4^ Health Care Research Center Nisshin Pharma Inc. Fujimino Japan

**Keywords:** comprehensive peptide analysis, food intake, ghrelin, structure–activity relationship, wheat peptide

## Abstract

Ghrelin is an endogenous orexigenic hormone mainly produced by stomach cells and is reported to influence appetite, gastrointestinal motility and growth hormone secretion. We observed that enzymatic digest of wheat gluten stimulated ghrelin secretion from mouse ghrelinoma 3‐1, a ghrelin‐releasing cell line. Further on, we characterized the ghrelin‐releasing peptides present in the digest by comprehensive peptide analysis using liquid chromatography–mass spectrometry and structure–activity relationship. Among the candidate peptides, we found that SQQQQPVLPQQPSF, LSVTSPQQVSY and YPTSL stimulated ghrelin release. We then named them wheat‐ghretropin A, B and C, respectively. In addition, we observed that wheat‐ghretropin A increased plasma ghrelin concentration and food intake in mice after oral administration. Thus, we demonstrated that wheat‐ghretropin stimulates ghrelin release both *in vitro* and *in vivo*. To the best of our knowledge, this is the first report of a wheat‐derived exogenous bioactive peptide that stimulates ghrelin secretion.

## Graphical abstract

By using peptide comprehensive analysis of the gluten digest and by peptide homology comparison with already known ghrelin‐releasing peptides, we discovered three novel peptides that we name wheat‐ghretropins A, B and C. We observed that wheat‐ghretropin A increased food intake and serum ghrelin levels after oral administration in mice. To the best of our knowledge, this is the first report of a wheat‐derived exogenous bioactive peptide that stimulates ghrelin secretion.

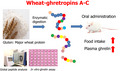

**Abbreviations**
GOAT: ghrelin *O*‐acyl transferase
MGN3‐1: mouse ghrelinoma 3‐1
nano‐LC–MS: nano‐liquid chromatography–mass spectrometry
PC1/3: prohormone convertase 1/3
pEL: pyroglutamyl leucine
SEM: standard error of the mean

Wheat (*Triticum aestivum*) is one of the three major grains and is used for a wide range of foods, such as bread, cakes, biscuits, pasta and noodles. Wheat is widely produced worldwide because it grows in many types of soil and weather conditions. Wheat contains approximately 10% protein, and the greatest amount is gluten. To date, a number of exogenous biologically active peptides mimicking endogenous molecules, such as hormones and neuropeptides, have been isolated from the enzymatic digests of wheat gluten. For example, opioid peptides, named gluten exorphin, were also isolated from the pepsin–thermolysin digest of gluten [1]. A hepatoprotective peptide, pyroglutamyl leucine, was identified in gluten hydrolysate [2].

The number of elderly people will continue to increase worldwide. Thus, a need for materials that improve the quality of life of the elderly is growing. It is known that appetite declines with age even in healthy elderly individuals, which is termed anorexia of aging [3]. A decrease in the food intake and consequent weight loss facilitate vulnerability to illnesses, such as malnutrition, frailty and sarcopenia, which impair quality of life. As such, an increased appetite will be beneficial for ameliorating anorexia.

Therefore, we focused on ghrelin, an endogenous orexigenic hormone from the stomach. Ghrelin is an endogenous 28‐amino acid peptide identified as an endogenous ligand for growth hormone secretagogue receptor [4, 5]. Ghrelin is encoded by the preproghrelin gene, which is activated after *n*‐octanoyl modification on the Ser3 residue by ghrelin *O*‐acyl transferase (GOAT) and processing by prohormone convertase 1/3 (PC1/3). It is known to stimulate food intake, gastric motility and growth hormone secretion [6, 7, 8]. Blood ghrelin levels are regulated in response to the body energy status; it increases during preprandial conditions or starvation and decreases postprandially. Plasma ghrelin levels are known to decrease [9, 10, 11], which may play a role in anorexia of aging. Thus, promoting ghrelin secretion may help to ameliorate the decline in food intake.

In our previous study, we revealed that a peptide derived from the enzymatic digest of soybean proteins, named soy‐ghretropin, increased ghrelin secretion [12]. In contrast, it is unknown whether wheat gluten and their derivatives affect ghrelin secretion. In this study, we found that the enzymatic digest of gluten using proteases stimulates ghrelin secretion in mouse ghrelinoma 3‐1 (MGN3‐1), a ghrelin‐releasing cell line [13]. We then investigated the ghrelin secretion‐stimulating peptide candidates present in the chymotrypsin digest based on the comprehensive analysis of the digest and similarity to ghrelin‐releasing dipeptides. We also examined the *in vivo* effects of ghrelin release‐stimulating peptides derived from gluten.

## Materials and methods

### Reagents

Peptides, including Ser‐Gln‐Gln‐Gln‐Gln‐Pro‐Val‐Leu‐Pro‐Gln‐Gln‐Pro‐Ser‐Phe (SQQQQPVLPQQPSF), Leu‐Ser‐Val‐Thr‐Ser‐Pro‐Gln‐Gln‐Val‐Ser‐Tyr (LSVTSPQQVSY) and Tyr‐Pro‐Thr‐Ser‐Leu (YPTSL), were chemosynthesized using the F‐moc strategy and purified by reverse‐phase high‐performance liquid chromatography. Sodium octanoate was purchased from Sigma‐Aldrich (St. Louis, MO, USA). Trypsin, chymotrypsin, pepsin and pancreatin were obtained from Sigma‐Aldrich, Seikagaku Corporation (Tokyo, Japan). Gluten was obtained from Nissin Pharma Corporation (Tokyo, Japan).

### Cell culture

MGN3‐1 cells, the ghrelin‐producing cell line from a gastric ghrelinoma from ghrelin promoter SV40‐T antigen‐transgenic mice, were cultured in DMEM supplemented with 10% fetal bovine serum, 100 U·mL^−1^ penicillin and 100 µg·mL^−1^ streptomycin at 37 °C in 10% CO_2_, as previously described [13].

### Enzymatic digests of wheat gluten

Gluten was dissolved in water at a concentration of 20 mg·mL^−1^. It was digested by trypsin or chymotrypsin (enzyme : substrate ratio = 1 : 100) at 37 °C for 5 h (pH 7.5). Similarly, pepsin digestion (enzyme : substrate ratio = 1 : 100) was performed at 37 °C for 5 h (pH 2.0). The pepsin pancreatin digestion was digested with pancreatin (enzyme : substrate ratio = 1 : 20) at 37 °C for 5 h (pH 7.5) after pepsin digestion. After boiling for 10 min to prevent the enzyme from digesting, the digests were freeze‐dried and stored at −20 °C until the experiments.

### Comprehensive peptide analysis of the enzymatic digest by the nano‐LC–MS

The peptides in wheat gluten digests were analyzed by nano‐liquid chromatography–mass spectrometry (nano‐LC–MS), followed by automatic sequence prediction. The nano‐LC–MS system consisted of an Easy nLC II nano‐flow liquid chromatography and Q Exactive high‐resolution mass spectrometer (Thermo Fisher Scientific, Inc., Waltham, MA, USA). Peptides were desalted on a GL‐Tip styrene divinylbenzene column (GL Science, Tokyo, Japan) according to the manufacturer's instructions and dissolved to 0.1 mg·mL^−1^ in 0.1% formic acid before nano‐LC–MS analysis. The desalted peptides were separated on a PepMap RSLC C18 capillary column (Thermo Fisher Scientific) using a gradient elution of 0.1% formic acid in water (solvent A) and 0.1% formic acid in acetonitrile (solvent B). The elution profile was configured as an isocratic elution of 5% of solvent B (0–5 min), a linear gradient of 5–35% solvent B (5–125 min), 35–80% solvent B (125–130 min) and 80% solvent B (130–140 min). The flow rate of the solvents and the column temperature were kept at 300 nL·min^−1^ and 40 °C, respectively.

The peptides were ionized by electrospray ionization and detected by the mass spectrometer at a resolution power of 70 000 and 17 500 for precursor mass scan and product mass scan, respectively. The sequences of peptides were predicted by Proteome Discoverer 2.3 (Thermo Fisher Scientific) using the mass spectrum obtained. The protein sequences used for the sequence prediction were obtained from the protein database in the National Center of Biotechnology Information using a search keyword of ‘*Triticum aestivum*’.

### Measurement of acylated ghrelin secretion in MGN3‐1 cells

Ghrelin secretion was measured using MGN3‐1 cells as described previously [12, 14]. Cells were seeded at 1 × 10^5^ cells/200 μL/well. After washing with Dulbecco's PBS, MGN3‐1 cells were exposed to peptide samples dissolved in DMEM containing 50 μm sodium octanoate at 37 °C for 4 h. The culture medium was collected and centrifuged at 800 ***g*** for 5 min at 4 °C. The supernatant was acidified with 10% (v/v) 1 N HCl and stored at –80 °C until analysis. These cellular supernatants were quantitatively analyzed using an acylated ghrelin enzyme immunoassay kit (Bertin Pharma, Montigny‐le‐Bretonneux, France) according to the manufacturer's protocol.

### Quantitative RT‐PCR

Cells were seeded at 1 × 10^5^ cells/200 μL/well. MGN3‐1 cells were incubated with test agents dissolved in DMEM containing 50 μm sodium octanoate at 37 °C for 4 h. Cells were corrected, and then total RNA was extracted from MGN3‐1 cells using the RNeasy Mini Kit (QIAGEN, Hilden, Germany). cDNA was generated by the Takara PrimeScript® RT Master Mix (Takara, Osaka, Japan). For quantitative real‐time PCR, we amplified the cDNA using the QuantStudio 1 (Thermo Fisher Scientific, Inc.) with THUNDERBIRD® qPCR Mix (Toyobo Co., Osaka, Japan) and each primer set specific for mouse β‐actin, preproghrelin, GOAT or PC1/3 according to the manufacturer's instructions (Table 1). The reactions were cycled 40 times with denaturation at 95 °C for 15 s and with annealing and elongation at 60 °C for 60 s. Normalized mRNA levels were expressed in arbitrary units obtained by dividing the averaged, efficiency‐corrected values for sample mRNA expression by that for β‐actin mRNA expression for each sample.

**Table 1:** Primer sets for measuring mRNA expression of genes associated with ghrelin synthesis.

Gene	Forward	Reverse
*β‐actin*	5′‐CTGCGCAAGTTAGGTTTTGTCA‐3′	5′‐TGCTTCTAGGCGGACTGTTACTG‐3′
*Preproghrelin*	5′‐CCCAGGCATTCCAGGTCAT‐3′	5′‐AACTGCAGATGGTGCCTGAAG‐3′
*GOAT*	5′‐CATGGGTCCCTACTCTCTGC‐3′	5′‐GGACTTCCTGTGGACTGAGC‐3′
*PC1/3*	5′‐GGCACCTGGACATTGAAAATTAC‐3′	5′‐TTCATGTGCTCTGGTTGAGAAGA‐3′

### *In vivo* ghrelin secretion

*In vivo* experiments were performed as previously described [12, 14]. Eleven‐week‐old male ddY mice were purchased from Japan SLC (Shizuoka, Japan). Mice were anesthetized, and blood was obtained from the orbital sinus 1 h after the oral administration of peptides dissolved in saline. Peptide solution was dosed at 0.1 mL per 10 g body weight. Then the blood was centrifuged at 1120 ***g*** for 10 min at 4 °C, and the supernatant was acidified with 10% v/v 1 N HCl before storing at −80 °C. The plasma ghrelin level was measured using the acylated ghrelin enzyme immunoassay kit.

### Food intake experiments

Mice were used for food intake experiments. Male ddY mice were obtained from the Japan SLC. All mice were maintained on a 12‐h light and 12‐h dark cycle condition (lights on 7 am–7 pm) and temperature‐controlled environment at 22–24 °C with *ad libitum* access to water and normal diet (CE‐2; CLEA Japan, Tokyo, Japan) or high‐fat diet (HFD, 60% kcal fat; Research diet, D12492). The care of all animals and procedures were approved by the Kyoto University animal committee. In the food intake measurement, 8‐week‐old male ddY mice were individually housed with free access to water and food pellets and were used after a 1‐week acclimation period. Food intake was measured at the indicated time periods after oral administration of wheat ghretropin A (0.3 mg·kg^−1^) or vehicle (saline) with feeding needle. For food intake experiment in light phase, the experiment was started at 10–11 am with fed condition. These were because long‐term fasting conditions might affect plasma ghrelin levels between groups, and fed condition makes a lower circulating ghrelin level in mice. For food intake experiment in dark phase, food was removed at 7 am, and the experiment was started at 7 pm with 12‐h fasting condition. Similarly, we performed food intake test in 4‐week HFD‐fed mice. After the experiment, all mice were euthanized by anesthesia overdose. All experiments were approved by the Kyoto University animal committee.

### Statistical analysis

The data are presented as mean ± standard error of the mean (SEM). Statistical analyses were performed using graphpad prism (GraphPad Software, San Diego, CA, USA) for a two‐tailed unpaired Student's *t*‐test, or one‐ or two‐way ANOVA followed by *post hoc* Tukey's, Dunnett's or Sidak's tests. A *P* value <0.05 was considered to be statistically significant.

## Results

### Chymotrypsin digest stimulated ghrelin secretion in the MGN3‐1 ghrelin‐releasing cells

**Fig. 1:**
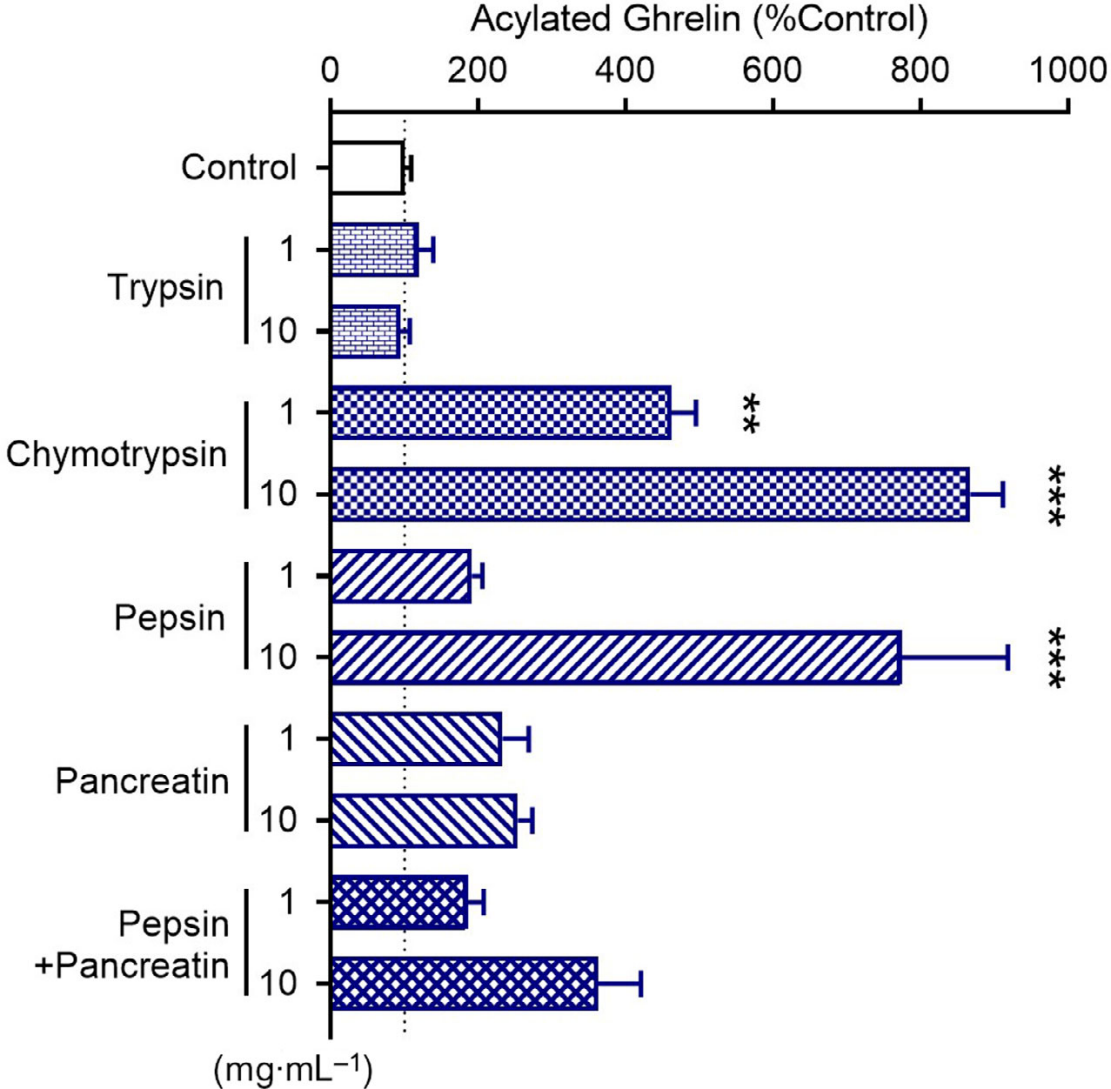
Effects of enzymatic digests of wheat gluten on ghrelin levels in the medium of the ghrelin‐releasing cell line MGN3‐1. The chymotrypsin and pepsin digests significantly increased the ghrelin levels. All digests at a dose of 1 or 10 mg·mL^−1^ were applied to the cells. **P < 0.01, ***P < 0.001 for one‐way ANOVA followed by Tukey's multiple comparisons tests. All error bars are SEM (*n* = 3–4).

We investigated the effects of enzymatic digests of wheat gluten on ghrelin secretion using a ghrelin‐releasing cell line, MGN3‐1. We used four enzymes: trypsin, chymotrypsin, pepsin and pancreatin. The ghrelin concentrations in the medium increased after incubation with the chymotrypsin or pepsin digest, suggesting that chymotrypsin and pepsin digests exhibit ghrelin‐releasing effects *in vitro* (Fig. 1). The ghrelin concentrations after application of the chymotrypsin digest were significantly higher than those after application of the pepsin digest at a dose of 1 mg·mL^−1^. We therefore focused on the chymotrypsin digest.

### Wheat‐ghretropins, novel ghrelin‐releasing peptides derived from wheat gluten

Next, we searched for ghrelin‐releasing peptides present in the digest. We performed comprehensive analysis of peptides in the chymotrypsin digest and detected 204 peptides in total. These peptides were screened by structural rules. We previously reported that information on the structure–activity relationship of dipeptides can be used to narrow down candidates for bioactive peptides [15]. We recently found that dipeptides with N‐terminal serine residues exhibit ghrelin‐releasing activity [16]. Among the detected peptides by the nano‐LC–MS, we therefore selected seven candidate peptides containing ghrelin‐releasing sequences at the C terminus and chemosynthesized them. Peptides sorted in order of peak intensity corresponding to rough peptide content are shown in Table S1.

**Fig. 2:**
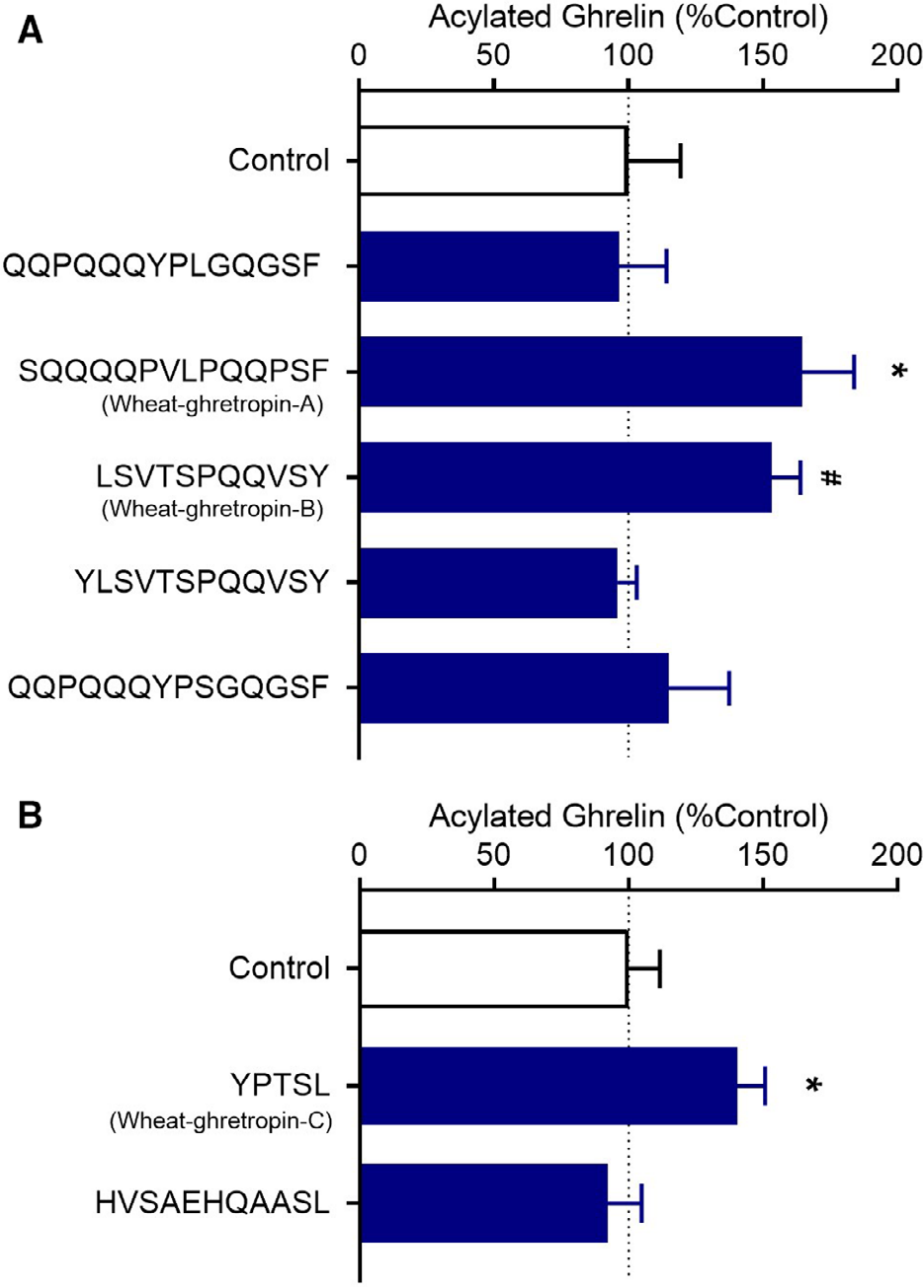
Discovery of novel ghrelin‐releasing peptides named wheat‐ghretropin. The ghrelin‐releasing activity of seven chemosynthesized peptide candidates was assessed. Peptides at a concentration of 1 mm were applied to MGN3‐1 cells. Effects of candidate peptides with Ser‐Phe (SF) and Ser‐Tyr (SY) at the C terminus (A) and Ser‐Leu (SL) at the C terminus (B) on ghrelin secretion. SQQQQPVLPQQPSF, LSVTSPQQVSY and YPTSL increased the ghrelin levels, and we named them wheat‐ghretropin A, B and C, respectively. ^#^ *P < *0.1, *P < 0.05 for one‐way ANOVA followed by Dunnett's multiple comparisons tests compared with the control medium group (A, B). All error bars are SEM (A, *n* = 3–4; B, *n* = 8).

**Fig. 3:**
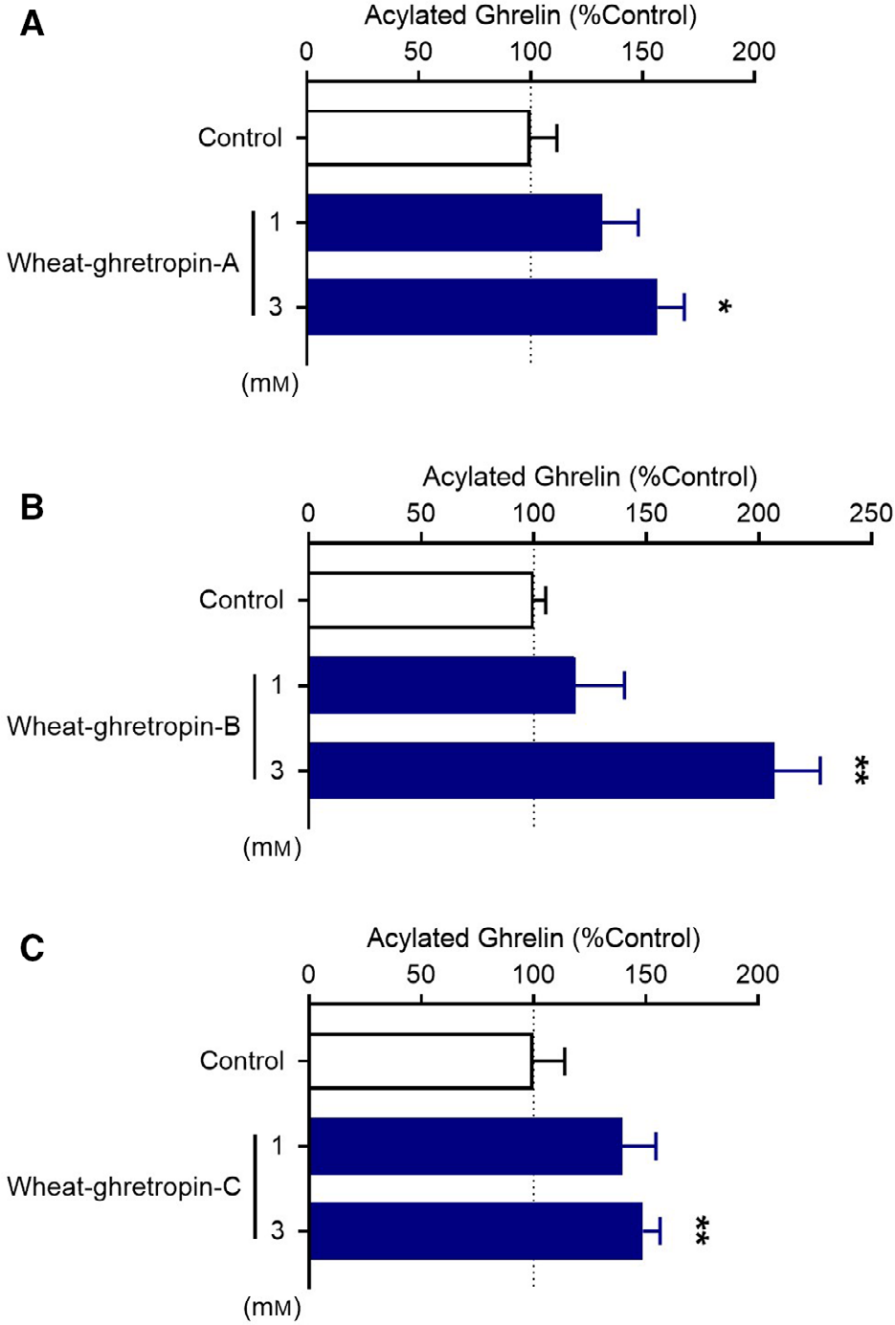
The ghrelin‐releasing activity of three chemosynthesized peptide candidates was assessed. Peptides at a concentration of 1 and 3 mm were applied to MGN3‐1 cells. Effects of wheat‐ghretropin A (A), wheat‐ghretropin B (B) and wheat‐ghretropin C (C) on ghrelin levels in the medium of MGN3‐1. Wheat‐ghretropin A, B and C significantly increased the ghrelin levels at a dose of 3 mm. *P < 0.05, **P < 0.01 for one‐way ANOVA followed by Dunnett's multiple comparisons tests compared with the control medium group (A–C). All error bars are SEM (A, *n* = 8; B and C, *n* = 4).

Among the candidates, SQQQQPVLPQQPSF, LSVTSPQQVSY and YPTSL increased ghrelin levels in the medium of MGN3‐1 (Fig. 2). Thus, we found ghrelin‐releasing peptides derived from a major protein of wheat. These peptides corresponded to glutenin low‐molecular subunit (81–94, 111–124) (accession number: CAA76890.1) and glutenin high‐molecular subunits (41–51) (accession number: AEO19857.1) and (280–284, 295–299, 694–698, 775–779) (accession number: CAA27052.1), respectively. Because these peptides increased ghrelin levels significantly at a dose of 3 mm, subsequent experiments were performed at this concentration (Fig. 3). We also confirmed that SQQQQPVLPQQPSF, LSVTSPQQVSY and YPTSL were detected in the chymotryptic digestion of gluten (1.39, 0.95 and 0.13 mg·g^−1^ digest, respectively). Thus, we named these ghrelin‐releasing peptides (SQQQQPVLPQQPSF, LSVTSPQQVSY and YPTSL) wheat‐ghretropin A, B and C, respectively.

**Fig. 4:**
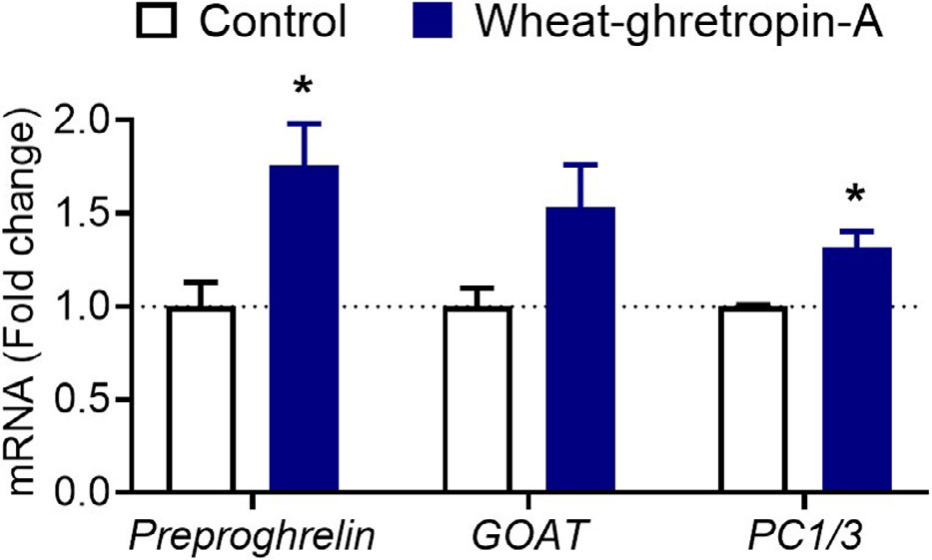
Effects of wheat‐ghretropin A on ghrelin synthesis in MGN3‐1 cells. Wheat‐ghretropin A (3 mm) was applied, and cells were collected 4 h later. The mRNA expressions of *preproghrelin*, *GOAT* and *PC1/3* were measured using quantitative PCR analyses. *P < 0.05 for *t* tests. All error bars are SEM (*n* = 3–4).

We next examined the mechanism underlying the ghrelin‐releasing activity of wheat‐ghretropin. The mRNA expression of preproghrelin, a ghrelin precursor, was increased by application of wheat‐ghretropin A. This result suggested that wheat‐ghretropin A promotes not only ghrelin secretion but also synthesis. Wheat‐ghretropin A also increased the mRNA expression of PC1/3, but not of GOAT (Fig. 4). Because wheat‐ghretropin B and C did not change the mRNA expression of these genes in this experiment, they might promote the ghrelin secretion rather than its synthesis (data not shown).

### Wheat‐ghretropin‐A increases plasma ghrelin levels and food intake *in vivo*

**Fig. 5:**
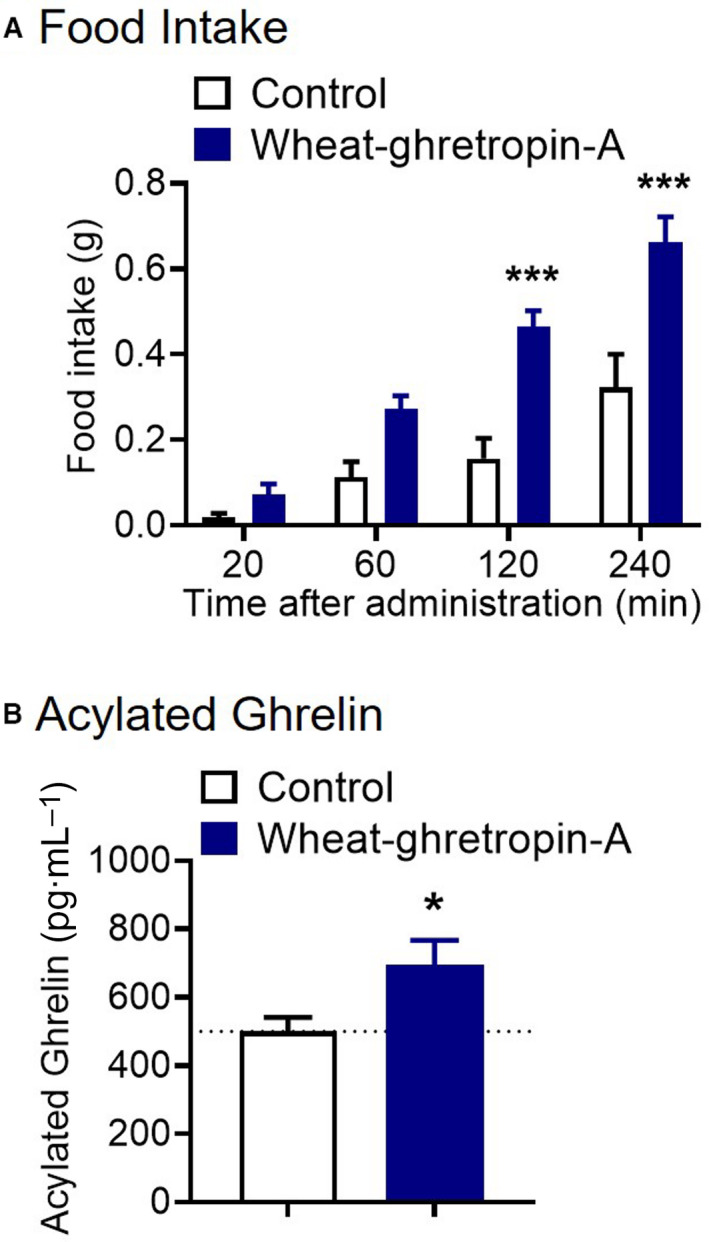
Oral administration of wheat‐ghretropin A increased the food intake and plasma acylated ghrelin levels in mice. (A) Wheat‐ghretropin A was administered orally at a dose of 0.3 mg·kg^−1^ body weight (0.03 mg·mL^−1^) to mice, and food intake was measured for 20–240 min. (B) Wheat‐ghretropin A was administered orally at a dose of 0.3 mg·kg^−1^ body weight (0.03 mg·mL^−1^) to mice, and plasma ghrelin levels were measured for 1 h. *P < 0.05, ***P < 0.001 for two‐way ANOVA followed by Sidak's multiple comparisons tests (A) or *t* tests (B). All error bars are SEM (A, *n* = 8; B, *n* = 20).

**Fig. 6:**
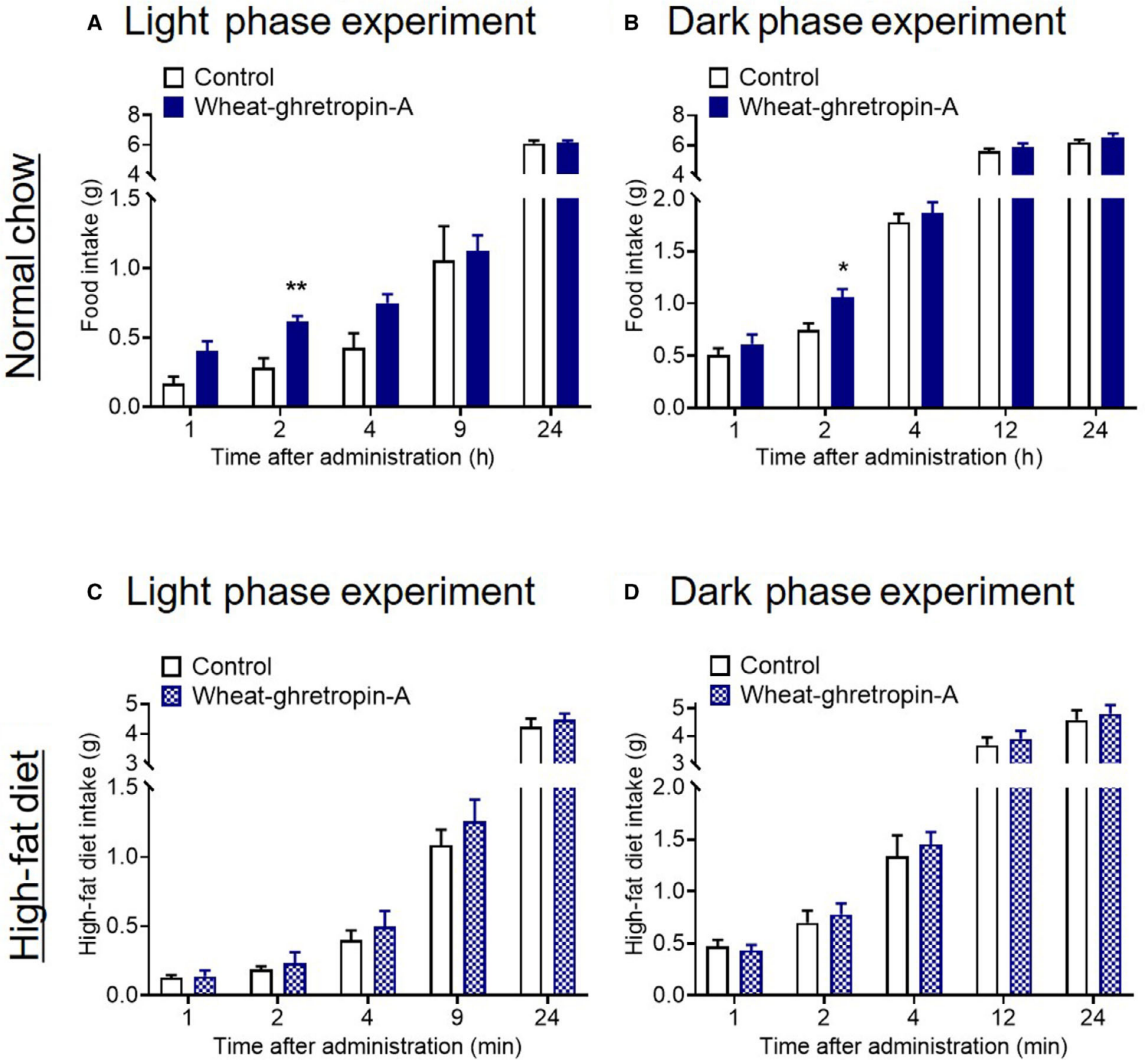
The orexigenic effect of wheat‐ghretropin A in normal‐chow‐ and HFD‐fed mice. (A, B) Wheat‐ghretropin A increased food intake in normal‐chow‐fed lean mice. Wheat‐ghretropin A was administered orally into non‐fasted mice at light phase (A) or 12‐h fasted mice at dark phase (B). (C, D) Wheat‐ghretropin A did not increase food intake in HFD‐fed mice at light phase (C) and dark phase (D) administration. *P < 0.05, **P < 0.01 for two‐way ANOVA followed by Sidak's multiple comparison test (A and B). All error bars are SEM (*n* = 8).

To assess whether wheat‐ghretropin affects the ghrelin system *in vivo*, we used male mice. Wheat‐ghretropin A increased the food intake after oral administration by oral gavage (Fig. 5A). Orally administered wheat‐ghretropin A also increased plasma ghrelin levels (Fig. 5B). To further investigate the effect of wheat‐ghretropin A *in vivo*, we performed oral administration of wheat‐ghretropin A into normal‐chow‐ and HFD‐fed mice and assessed the orexigenic effect in light and dark phases. We found that wheat‐ghretropin A shows an orexigenic effect in both light phase and dark phase injection into normal‐chow‐fed mice (Fig. 6A,B). Importantly, we did not observe these orexigenic effects of wheat‐ghretropin A in HFD‐fed mice under light and dark phase experiment (Fig. 6C,D). Although a single administration of wheat‐ghretropin A showed orexigenic effects in lean mice, 7‐day continuous administration of wheat‐ghretropin A (once a day for 7 days) did not significantly increase food intake and body weight in normal‐chow‐fed lean mice (Fig. S1). Thus, we demonstrated that orally administered wheat‐ghretropin A acutely shows orexigenic functions *in vivo*.

## Discussion

We found that enzymatic digests of wheat gluten stimulated ghrelin secretion. Based on comprehensive peptide analysis of the chymotrypsin digest and information on ghrelin‐secretory dipeptides, we found that SQQQQPVLPQQPSF, LSVTSPQQVSY and YPTSL stimulate ghrelin secretion, and named them wheat‐ghretropin A, B and C, respectively. We also demonstrated that wheat‐ghretropin A stimulates ghrelin release and food intake *in vivo*.

Wheat‐ghretropin A stimulated food intake stronger than peptides previously found, although it has a longer chain, which was assumed to be disadvantageous for absorption. This suggests a relationship between peptide ghrelin secretion and chain length. In this study, we revealed that gluten pepsin digests increased ghrelin levels more than pepsin‐pancreatin digests. This suggests that peptides whose chain has been shortened by pancreatin digestion have weaker ghrelin‐releasing effects. In addition, a comprehensive analysis of dipeptides revealed that many of them suppressed the secretion of ghrelin, suggesting that small peptides often suppress ghrelin secretion [16]. Furthermore, we previously found that the other medium‐molecular‐weight peptides exhibited more potent activity than short‐chain peptides [12, 14]. Although smaller peptides derived from the parent tridecapeptide, wheat‐ghretropin A, after degradation by gastrointestinal proteases being absorbed cannot be excluded, wheat‐ghretropin A may need a long chain to affect food intake.

As previously reported, ghrelin‐releasing cells are morphologically classified into closed and open types [17]. It is known that the closed‐type cells, present predominantly in the stomach, sense glucose or endogenous hormones in the blood, whereas the open‐type cells, present in the lower gastrointestinal tract, may sense the luminal content. Thus, we hypothesized that orally administered wheat‐ghretropin A acts on open‐type cells and stimulates ghrelin secretion. Taken together, wheat‐ghretropin A may increase food intake through gut–brain communication.

In MGN3‐1, a ghrelin‐releasing cell line, wheat‐ghretropin A increased the mRNA expression of preproghrelin and PC1/3, suggesting that it affects the ghrelin synthesis process. In contrast, previously discovered ghrelin‐releasing peptide derived from other food proteins, including soy‐ghretropin, did not change the mRNA expression related to the ghrelin synthesis process [12]. Wheat‐ghretropin A is the first example of a food‐derived peptide that increases the mRNA expression of preproghrelin. Thus, wheat‐ghretropin A may promote ghrelin secretion through novel pathways from other peptides.

In this study, we found wheat‐ghretropins from gluten, known as a major wheat protein. We also found soy‐ghretropin derived from soy major proteins, β‐conglycinin, implying food‐derived peptides sometimes alter ghrelin secretion and food intake after oral administration. There may be common structural rules for the exertion of ghrelin‐releasing activities. Ser residues are present at the second or third position from the C terminus. If the detailed structure–activity relationship is elucidated, it may be highly beneficial in searching for novel ghrelin‐releasing peptides from food proteins. In the future, the elderly population will increase, and foods stimulating ghrelin signaling may be helpful for elderly people with anorexia of aging.

Our *in vivo* study revealed a previously unidentified link between wheat‐derived peptides and food intake. In addition to its effect on ghrelin secretion *in vivo*, wheat‐ghretropin A resulted in significantly increased food intake after oral administration in light and dark phases in young lean mice. Also, 7‐day continuous administration of wheat‐ghretropin A did not significantly increase food intake and body weight in normal‐chow‐fed lean mice. This may be because of the acute orexigenic effect of wheat‐ghretropin A. Consistently, 24‐h cumulative food intake was not significantly altered after wheat‐ghretropin A administration. Further studies will thus need to clarify whether long‐term administration of wheat‐ghretropin A affects food intake and body weight in lean mice and/or anorexia animal models. Most importantly, in HFD‐fed mice, wheat‐ghretropin A did not show the orexigenic effect after oral administration, suggesting that wheat‐ghretropin A may not accelerate the diet‐induced obesity.

In conclusion, we found three novel ghrelin‐releasing peptides from the enzymatic digest of wheat gluten and named them wheat‐ghretropin A, B and C. We also demonstrated that wheat‐ghretropin A stimulates ghrelin secretion *in vitro* and *in vivo*, and increased the food intake after oral administration. This is the first report of ghrelin release stimulated by peptides from wheat gluten.

## Conflict of interest

The authors declare no conflict of interest. MM and HI are employed by Nisshin Pharma Inc.

## Author contributions

KO conceived and supervised the study. KT, KK, SA, SO and JN performed the experiments and analyzed the data with some help from YT, H Iwakuwa, and SM. KI, MS, AK and HS performed comprehensive peptide analysis. MM and H Ikemoto contributed to the sample preparation. KT, KK and KO wrote the manuscript. All authors discussed the results and the manuscript, and approved the final version of the manuscript.

## Supporting information


**Fig. S1.** The effect of 7‐day continuous administration of wheat‐ghretropin A. Male mice were maintained on normal chow and injected with wheat‐ghretropin A (0.3 mg/kg, once per day, p.o.) or vehicle (saline) during the indicated period. Body weight (A) and food intake (B) were measured every day. Age‐ and body weight‐matched cohorts were used (*n* = 8/group).

**Table S1.** Detected peptides from comprehensive LC–MS analysis of the gluten chymotrypsin digest.

## Data Availability

The datasets generated during and/or analyzed during this study are available from the corresponding author on reasonable request.
