# Supplementary material for: Wheat‐ghretropins: novel ghrelin‐releasing peptides derived from wheat protein
Source: FEBS Open Bio. 2021 Mar 18;11(4):1144–52. doi: 10.1002/2211-5463.13124 (PMC8016139; doi:10.1002/2211-5463.13124)
Supplement: Supplementary file 1 — Fig. S1. The effect of 7‐day continuous administration of wheat‐ghretropin A. Male mice were maintained on normal chow and injected with wheat‐ghretropin A (0.3 mg/kg, once per day, p.o.) or vehicle (saline) during the indicated period. Body weight (A) and food intake (B) were measured every day. Age‐ and body weight‐matched cohorts were used (n = 8/group). [file FEB4-11-1144-s002.pdf]

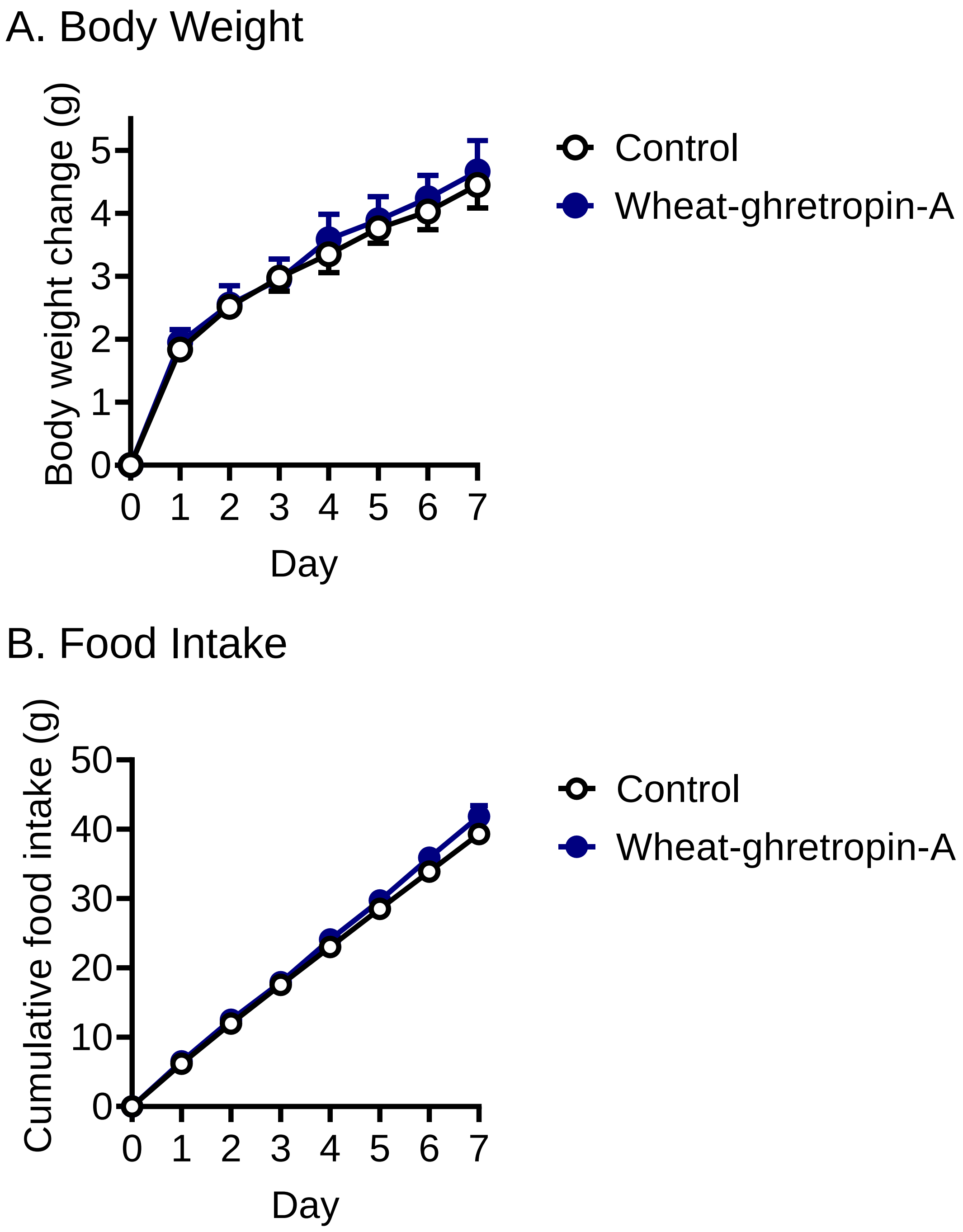

Supplementary Figure 1.

The effect of 7 days-continuous administration of wheat-ghretropin-A. Male mice were maintained on a normal-chow and injected with wheat-ghretropin-A (0.3 mg/kg, once per day, p.o.) or vehicle (saline) during the indicated period. Body weight (A) and food intake (B) were measured every day. Age- and body weight matched cohorts were used (n = 8/group).
