# Supplementary material for: Wheat‐ghretropins: novel ghrelin‐releasing peptides derived from wheat protein
Source: FEBS Open Bio. 2021 Mar 18;11(4):1144–52. doi: 10.1002/2211-5463.13124 (PMC8016139; doi:10.1002/2211-5463.13124)
Supplement: Supplementary file 2 — Table S1. Detected peptides from comprehensive LC–MS analysis of the gluten chymotrypsin digest. [file FEB4-11-1144-s001.pdf]

Supplementary table 1. Detected peptides from comprehensive LC/MS analysis of the gluten chymotrypsin digest.

Sequence	Intensity	R.T. [min]	m/z	Charge	Score	Modifications
VRVPVPQLQPQNPSQQQPQEQVPL	1.30E+10	63.11	912.16	3	5.10	
SHIPGLERPSQQQPLPPQQTL	2.76E+09	50.75	784.42	3	4.29	
LQLQPFPQPQLPY	2.54E+09	81.98	784.93	2	3.68	
FQPSQQNPQAQGF	2.29E+09	41.02	738.85	2	2.63	
VRVPVPQLQPQNPSQQQSQEQVPL	1.62E+09	60.71	908.82	3	5.04	
GIPALL	1.47E+09	65.02	583.38	1	1.26	
SLARSQML	1.26E+09	29.79	453.25	2	1.97	
RPQQPYPQPQPQY	1.20E+09	33.70	813.90	2	3.10	
SHHQQQQPIQQQPQPF	1.17E+09	30.54	652.65	3	4.81	
QQPQQQYPSGQGSFQPSQQNPQAQGF	1.03E+09	50.09	964.11	3	5.57	
QQPQQQYPLGQGSF	7.38E+08	53.33	803.39	2	3.94	
AQGTF	7.38E+08	15.89	523.25	1	1.44	
EQTVVPPKGGSFYPGETTPL	6.96E+08	57.84	1052.53	2	2.97	
LGQQQPFPPQQPYPQPQPFPSQQPY	6.69E+08	68.79	974.81	3	4.15	
GQQPQQQQLAQGTF	6.00E+08	41.84	779.88	2	3.37	
LTSPQQLGQGQQPRQW	4.88E+08	43.93	926.48	2	4.03	
SLARSQML	4.65E+08	21.19	461.24	2	1.27	1xOxidation [M7]
LSVTSPQQVSY	4.43E+08	47.44	604.81	2	2.06	
QQQPPFLQQQQPSLPQQPPFSQQQQQL	4.40E+08	72.49	1067.88	3	5.40	
QQSGQGQQGHYPTSLQQPGQGQQGHY	3.80E+08	32.35	942.10	3	5.51	
YLTSPQQSGQW	3.79E+08	49.30	647.81	2	2.57	
YLTSPQQLGQGQQPRQW	3.23E+08	49.99	672.34	3	4.93	
PSQQQPQEQVPL	3.22E+08	40.65	689.85	2	2.54	
GVGTGVGS	3.14E+08	14.42	633.32	1	2.00	
QQQLVLPQQQIP	3.04E+08	64.62	710.40	2	2.34	
HVSAEQQAASPM	2.89E+08	13.02	636.29	2	3.04	1xOxidation [M12]
YPGQASSQRPGQGQQPGQGQQEY	2.87E+08	29.82	826.05	3	4.60	
HVSAEHQAASL	2.72E+08	18.17	383.86	3	2.64	
SQQQQPVLPQQPSF	2.62E+08	55.75	806.41	2	2.99	
YLSVTSPQQVSY	2.57E+08	54.05	686.34	2	2.58	
QQGYYPTSPQQLGQGQQPRQW	2.37E+08	51.44	825.73	3	5.03	
TIAPF	2.32E+08	48.51	548.31	1	1.00	
VRVPVPQLQPQNPSQQQPQEQVPVVQQQQF	2.21E+08	64.27	1160.28	3	4.49	
YPGQASSQRPGQGQQEY	2.13E+08	28.05	940.93	2	4.46	
LQPHQIAQLEVM	2.09E+08	55.11	703.88	2	1.49	
QQPQQQYPSGQGSF	2.07E+08	37.50	790.36	2	3.58	
QQGYYPTSPQQLGQGQQPGQW	2.06E+08	61.06	1188.55	2	3.48	
RPQQPYPQPQPQ	1.94E+08	23.87	732.37	2	2.99	
VQPQQLPQFEEIRNL	1.93E+08	67.25	613.66	3	4.15	
QQSGQGQQGYYPTSPQQSGQGQQPGQW	1.77E+08	49.32	979.11	3	4.44	
QQPGQWQQPGQGQPGYY	1.72E+08	48.90	973.94	2	3.47	
QSGQGQPGYY	1.64E+08	26.18	542.74	2	1.55	
LALQTLPAI	1.46E+08	47.24	470.30	2	0.42	
VRVPVPQ	1.28E+08	30.72	397.75	2	1.17	
YPGQASPQQPGQGQQPGKW	1.28E+08	37.56	680.33	3	3.56	
SQQQLGQQPQQQQL	1.27E+08	34.02	819.91	2	3.21	
QQPQQQYPSGQGSFQPSQQN	1.21E+08	35.73	754.68	3	3.38	
VHPSIL	1.12E+08	30.57	665.40	1	1.24	
SVTSPQQVSYYPGQASSQRPGQGQQEY	1.11E+08	44.66	986.46	3	2.37	
QLPPFSQQQSPF	1.01E+08	66.37	702.35	2	1.65	
SQPQHPISQQQQQQQQQQQEQQIL	9.55E+07	35.39	1014.50	3	4.68	
LQPHQIAQL	8.21E+07	39.05	524.30	2	2.31	
QLGQQPQQQQL	8.14E+07	33.31	648.34	2	3.17	
AQGTFLQPHQIAQLEVM	7.94E+07	69.50	955.99	2	0.50	
YPTSL	7.40E+07	37.45	580.30	1	1.53	

The top 55 are indicated.
